# The Stellate Ganglion Block for PTSD: A Retrospective Clinical Case Series

**DOI:** 10.3390/ijerph23060758

**Published:** 2026-06-05

**Authors:** Michael Hollifield, Jennifer Lai-Trzebiatowski, Michael Alkire, Tyler C. Smith, Christine J. Eickhoff, Nima Fahimian, Rostam Khoshsar, Rajika Tobey, Staci Becker, Rossean C. Rossel, Sarah Madison, Patrick Wu, Amy Treadwell, Christopher Reist

**Affiliations:** 1 Tibor Rubin VA Medical Center, 5901 E. 7th St, Long Beach, CA 90822, USA; laijenniferk@gmail.com (J.L.-T.); michael.alkire@va.gov (M.A.); nima.fahimian@va.gov (N.F.); drkhoshsar@biohealthpain.com (R.K.); rajika.tobey@va.gov (R.T.); staci.becker@va.gov (S.B.); rossean.rossel@va.gov (R.C.R.); sjmadisonmd@gmail.com (S.M.); patrick.wu@va.gov (P.W.); amy.treadwell@va.gov (A.T.); creist@uci.edu (C.R.); 2 Department of Psychiatry and Behavioral Sciences, George Washington School of Medicine & Health Sciences, Washington, DC 20052, USA; 3 The War Survivors Institute, 5813 E. 2nd Street, #637, Long Beach, CA 90803, USA; 4 Department of Health Services and Leadership, School of Health Professions, National University, San Diego, CA 92107, USA; tsmith@nu.edu; 5 The Veterans Health Administration, National Center for Healthcare Advancement and Partnerships, Washington, DC 20420, USA; christine.eickhoff@va.gov; 6 BioHealth Pain Management, 15901 Hawthorne Blvd., Suite 240, Lawndale, CA 90260, USA; 7 Anesthesia Associates Medical Group, 5276 Hollister Avenue, Suite 308, Santa Barbara, CA 93111, USA; 8 Science 37, 800 Park Office, Suite 3606, Durham, NC 27709, USA

**Keywords:** posttraumatic stress disorder, PTSD, stellate ganglion block, neuromodulation

## Abstract

**Highlights:**

**Public health relevance—How does this work relate to a public health issue?**

- Posttraumatic Stress Disorder (PTSD) is an environmentally caused illness that has a community lifetime prevalence of approximately 7% (U.S.), confers a significant risk for other medical illnesses, co-morbid psychiatric disorders, and behavioral manifestations, and incurred excess costs of approximately $232B in 2018.
- This is a retrospective clinical case series from a large healthcare system that reports on the rapid and sustained reductions in PTSD and anxiety symptoms using the Stellate Ganglion Block (SGB) procedure.

**Public health significance—Why is this work of significance to public health?**

- Even a 30% sustained reduction in PTSD symptoms might save significant individual morbidity and excess costs.
- This is the largest clinical case series to date that shows clinically meaningful, rapid, and sustained symptom reductions of ≥30% in 60% to 67% of patients treated with the SGB.

**Public health implications—What are the key implications or messages for practitioners, policy makers and/or researchers in public health?**

- The SGB appears to be effective for achieving rapid and sustained clinically meaningful reductions of PTSD and anxiety symptoms for a majority of patients in a real-world clinical setting in a large healthcare system for veterans.
- Theoretically, the rapid and sustained symptom reduction associated with SGB may reduce risks associated with PTSD including suicide, co-morbid psychiatric disorders, and other medical illnesses. Future research needs to test this theory and determine benefits on public health markers in addition to individual ones.

**Abstract:**

Background/Objectives: Extant data suggest that the Stellate Ganglion Block (SGB) is effective for posttraumatic stress disorder (PTSD). Clinical data from a large healthcare system are lacking. We report data from a clinical project in the Veterans Health Administration. Methods: Retrospective data of PTSD and anxiety for 579 patients who received one or more SGBs were analyzed on the full sample and on those who had complete data using general linear models. Results: Receiving the first SGB provided a 36% and 30% reduction in PTSD symptom scores at 1-week and 1-month post-SGB, respectively. Those who received 2–4+ SGBs showed lower pre-SGB PTSD symptom scores and trends for lower scores at 1-week and 1-month post-SGB. Overall, 78% and 71% of patients had a reliable (Δ ≥ −5 points) change and 68% and 60% had a clinically meaningful (Δ ≥ −10 points) change in PTSD symptom scores from pre-SGB to 1-week and 1-month post-SGB, respectively. There were clinically meaningful reductions for anxiety in 51.5% and 48.3% at 1-week and 1-month post-SGB, respectively. Conclusions: These data corroborate existing data about the benefit of SGB for PTSD and anxiety and are unique in showing an association between repeat SGBs and lower PTSD symptoms at subsequent baseline.

## 1. Introduction

Posttraumatic Stress Disorder (PTSD) is a debilitating disorder characterized by re-experiencing aspects of the original trauma, avoidance of trauma reminders, negative alterations in cognition and mood, and hyperarousal [1]. Lifetime prevalence of PTSD in U.S. community samples is about 6.8% [2] and as high as 30% in Vietnam veterans [3] and rape survivors [4]. PTSD also is a risk factor for suicide and serious medical illness throughout life [5]. Excess costs were estimated at $232B in the U.S. in 2018 [6].

Evidence-based treatments for PTSD have both value and limitations. Pharmacotherapy trials have shown significant yet clinically modest reductions on the Clinician-Administered PTSD Scale [7,8]. Various psychotherapies provide significant treatment effects [9], yet they can be associated with high rates of treatment non-engagement and dropout [9], prompting the field to continue searching for other effective and tolerable treatment approaches [10]. Evidence points to advantages of somatic and non-trauma focused approaches, such as imagery [11], mindfulness [12], and acupuncture [13,14,15].

Stellate Ganglion Blocks (SGBs) have historically been used to treat complex regional pain syndromes, causalgia (nerve injury), and intractable angina. While the local anesthetic effect remains for less than 24 h, the clinical effect can last for months. SGBs provide therapeutic benefit for what are postulated to be sympathetically mediated syndromes [16]. SGBs have been shown to be effective for PTSD in case studies [17,18,19,20] and case series [21,22,23,24,25,26]. There are two published randomized controlled trials (RCTs) of SGBs for PTSD with mixed results—the first demonstrating non-statistically significant results [27] while the second showed positive and statistically significant effects of SGB vs. a sham procedure [28].

Through a partnership with the Veteran Health Administration’s (VA) National Center for Healthcare Advancement and Partnerships (HAP) beginning in 2017, we developed the SGB for PTSD Innovation Program (SPIP), a collaborative clinical project serving Veterans from five VA Hospitals in the southwest U.S. The goals of the project were to develop infrastructure, policies, and procedures and collect data about the multidisciplinary execution of using SGBs for PTSD treatment in the VA. Since the SPIP’s inception, over 1600 SGBs were conducted. This report is part of a dissemination effort and presents clinical data and a brief discussion of the infrastructure/policies and procedures that were developed as the program was first implemented and evolved. The clinically important questions we aimed to answer with the data were as follows: (1) What were the effects on PTSD on a population basis? (2) Were there differences in effects on PTSD between those with one vs. more than one treatments? (3) What percentage of patients had a reliable (Δ ≥ −5 points) and clinically meaningful (Δ ≥ −10 points) PTSD symptom reduction at 1-week and 1-month post SGB? (4) What effect do SGBs have on anxiety?

## 2. Materials and Methods

### 2.1. Infrastructure Development

The SGB clinical enterprise began at the Long Beach VA with a pilot study (2012–2016) [23] followed by the partnership with HAP from 2017 through September 2024. Both periods were part of the development process of the SPIP, which included personnel and team building, acquisition of space, provisions for dedicated clinician time, logistics management, and iterative process improvement. The SPIP continues as of this writing, supported by the Long Beach VA.

### 2.2. Policies and Procedures (P/P)

The P/P were co-developed with the infrastructure and governed mental health functions, surgical/anesthesiology functions, service-line agreement(s), and memoranda of understanding between participating VA sites.

### 2.3. Patients and Project Design

This is a retrospective review and analysis of SPIP data for the period 21 September 2018 to 23 February 2024. Patient enrollment criteria in the SPIP included being a veteran at the Long Beach VA or at a VISN 22 VA with whom the SPIP had an active memorandum of understanding (MOU), having a diagnosis of PTSD from any trauma type, and having had at least two evidence-based practice (EBP) treatment trials for PTSD or having failed such trials due to side-effects causing withdrawal from treatment. Patients were excluded from the SPIP if they had a psychotic episode in the previous 6 months, a chronic psychotic disorder, active moderate or severe substance use disorder, uncontrolled thyroid disease, moderate or severe untreated sleep apnea, and other medical risks such as abnormal neck morphology, cardiac arrhythmia, severe traumatic brain injury, or conditions deemed as contraindications to a SGB by the medical team. Judicious medical decisions were made on any case that required a review to ensure patient safety.

### 2.4. Procedures

Patients were referred by any licensed independent practitioner (LIP) within the VA, governed by the criteria for the SPIP noted above. All patients received a psychiatric diagnostic examination for program inclusion and exclusion and a pre-operative review by the anesthesiology team, and were asked to complete the instruments noted below. Patients were asked if they would like to have additional therapy as part of the SPIP, including psychotherapy, acupuncture, or biofeedback, or if they wanted to continue care with their referring clinician. The clinic-registered nurse coordinator collaborated with the Health Administration Service to schedule and arrange appointments and medical follow-up. Patients had the option to request repeat SGBs at 3-month intervals; at each request they received psychiatric re-evaluation to determine if they had received benefits to warrant the SGB and continue to qualify for the program.

The SGB consisted of injecting 8 to 10 cc of 0.5% ropivacaine deep to the prevertebral fascia at the ventral aspect of the longus colli muscle, medial to Chassaignac’s tubercle under ultrasound guidance, after consent and under sterile conditions. Anesthetic volume varied dependent on the spread visualized as hypoechoic (dark) space occupying “matter.” After 5 min the patient was examined, documenting their subjective emotional/anxiety rating on a scale of 0–10 and a Horner’s syndrome rating (2 points for each of the following: ptosis, miosis, scleral injection/redness; total of 6) [29]. For patients with a Horner’s rating less than 5, an immediate repeat injection up to 5 cc of anesthetic is offered. The patient is then monitored with vital signs for 30 min and is released home with an adult chaperone.

The additional therapies of psychotherapy, acupuncture, or biofeedback were delivered clinically, with most patients receiving 12 sessions of each, though there was variability dependent on clinician–patient discussion.

Efforts were made to collect clinical data pre-SGB, immediately post-SGB (within 10 min), and 1-week, 1-month, and 4-months post-SGB. Four-month data are not included in this report due to the low percentage of complete data from this time point.

### 2.5. Measures

The PTSD Checklist for DSM-5 (PCL-5) [30] is a 20-item self-rating scale that assesses the DSM-5 Criteria B-E symptoms of PTSD. Respondents rate the degree to which each symptom bothered them during the past week or month on an ordinal scale ranging from not at all (0) to extremely (4) with a total range from 0 to 80. Reductions of ≥5 points and ≥10 points are considered “reliable” and “clinically meaningful” changes, respectively. The PCL-5 was administered at four time points, including pre-SGB, 1-week, 1-month, and 4-months post-SGB. The “past week” version was administered at the 1-week post-SGB timepoint and the “past month” version was administered at all other time points.

The Generalized Anxiety Disorder-7 (GAD-7) [31] is an anxiety scale with good reliability and validity. The initial scale metrics showed an internal consistency of α = 0.92 and good test-retest reliability (intraclass correlation = 0.83). Respondents rate the degree to which they have been bothered in the past 2 weeks on an ordinal scale from 0 (Not at all) to 3 (nearly every day) with a total range of 0–21. Increasing scores on the scale are strongly associated with multiple domains of functional impairment. Criteria have been identified that optimize sensitivity (89%) and specificity (82%) and the scoring is as follows: 0–4 is minimal anxiety, 5–9 is mild anxiety, 10–14 is moderate anxiety, and 15–21 is severe anxiety. A reduction of ≥ 6 points with a change in severity rating is considered “clinically significant.” The GAD-7 was administered at the same four time points as the PCL-5. The GAD-7 administration was started later in the project so there are less GAD-7 than PCL-5 data.

### 2.6. Data Reduction and Analyses

A database was created containing all information about patients, contact dates, procedure dates, questionnaire scores and dates, and clinical notes that helped personalize contact and intervention. Data were de-identified for analyses. Bivariate and group analyses (*t*-tests and chi-square tests) were conducted to identify differences in age and gender with respect to the number of treatments received. To assess the effects of SGBs on symptoms over time, paired *t*-tests were used. Ordinary least squares and analysis of variance were used to assess effects of SGBs over time by group, with the four groups of having had (1) only one SGB, (2) only two SGBs, (3) only 3 SGBs, and (4) four or more SGBs. Statistical significance was set at the *p* < 0.05 level.

## 3. Results

Table 1 describes gender, age, and the number of patients by number of SGBs received during the project. Five-hundred and seventy-nine unique patients received 1251 SGBs. There were no differences between number of SGBs received on sex or age.

### 3.1. Effects on PTSD Symptom Scores on a Population Basis

**Figure 1:**
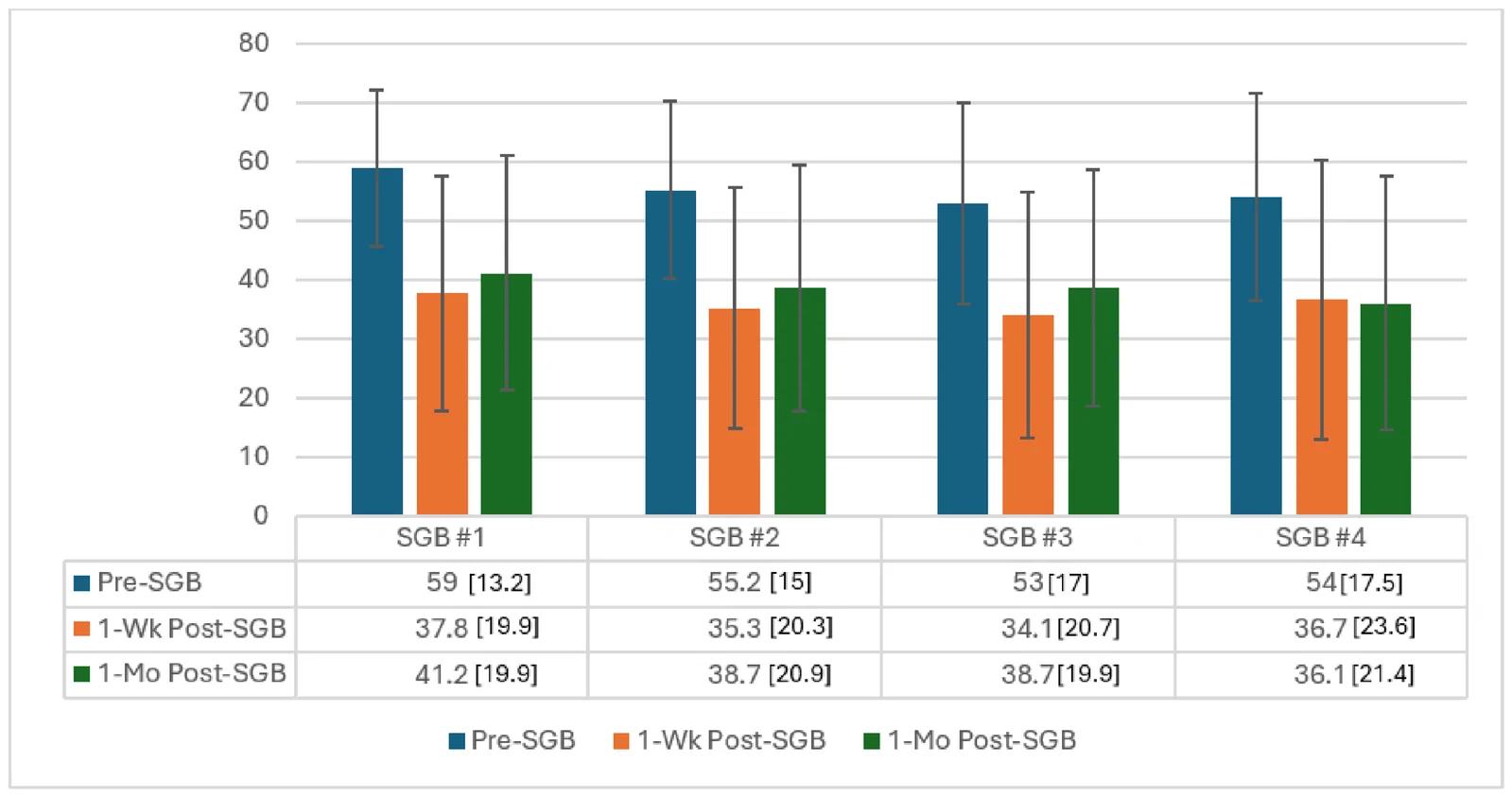
Pre- and post-PCL-5 scores by number of SGB treatments.

Of the 579 veterans that received their first SGB treatment, the PCL-5 was completed by 501, 339, and 327 veterans at pre-SGB, 1-week post-SGB, and 1-month post-SGB, respectively. There was a significant effect from pre- to 1-week post- (Δ = −21.2 [19.0], t = 20.8, *p* < 0.001, 36%Δ) and pre- to 1-month post-SGB (Δ = −17.8 [17.5], t = 19, *p* < 0.001, 30%Δ). Figure 1 shows PCL-5 scores by time for SGB treatments #1–4+ on a population basis (PCLs are not matched by individuals).

### 3.2. Comparisons Between Number of SGBs in Those with Complete PTSD Symptom Data

Data for these analyses were used for those who completed the PCL-5 at all three time points at each SGB. Table 2 summarizes data of those patients who received only 2 blocks, only 3 blocks, and 4+ blocks. It shows data from the terminal block as well as previous blocks. Mean scores were significant for time (MANOVA: Wilks 0.43, *F* = 121.52_[2,185]_, *p* < 0.0001) but not time × group (MANOVA: Wilks 0.99, *F* = 0.43_[6,370]_, *p* = 0.86), meaning there were no between-group differences from pre-SGB through 1-month post-SGB. To answer this question another way, we compared data from SGB #1 to SBG #2–4 combined for each time point. For pre-SGB data, there was a significant difference contrasting #1 with #2–4 (*LMS* = 1017.55, *F* = 4.96, *p* < 0.03), with the combined #2–4 group having lower PCL-5 scores. There were strong trends for contrast differences at 1 wk post-SGB (*LMS* = 1470.98, *F* = 3.31, *p* = 0.07) and at 1 mo post-SGB (*LMS* = 1504.00, *F* = 3.57, *p* = 0.06), also favoring lower scores in the combined #2–4 group. Mean PCL-5 reduction pre-SGB to 1 mo post-SGB by group for those that had complete PCL-5 data at both time points was group 1, *n* = 118, Δ = −15.6 [16.2], group 2, *n* = 44, Δ = −14.3 [17.6], group 3, *n* = 22, Δ = −14.5 [19.0], and group 4, *n* = 39, Δ = −20.6 [17.9], within group all *p* < 0.001, between group NS. Percent of absolute symptom reduction on the PCL−5 ranged from 31.8% (after SGB #2 for those who had only 3 SGBs) to 42% (after SGB #1 for those who had only 3 SGBs) after 1 week and from 22.3% (after SGB #3 for those who had only 3 SGBs) to 41.3% (after SGB #1 for those who had only 3 SGBs) after 1 month. Correlations between number of SGBs and PCL-5 scores were *n*= 254, *r* = 0.06, *p* = 0.37 at 1 week and *n* = 223, *r* = 0.11, *p* = 0.10 at 1 month.

**Figure 2:**
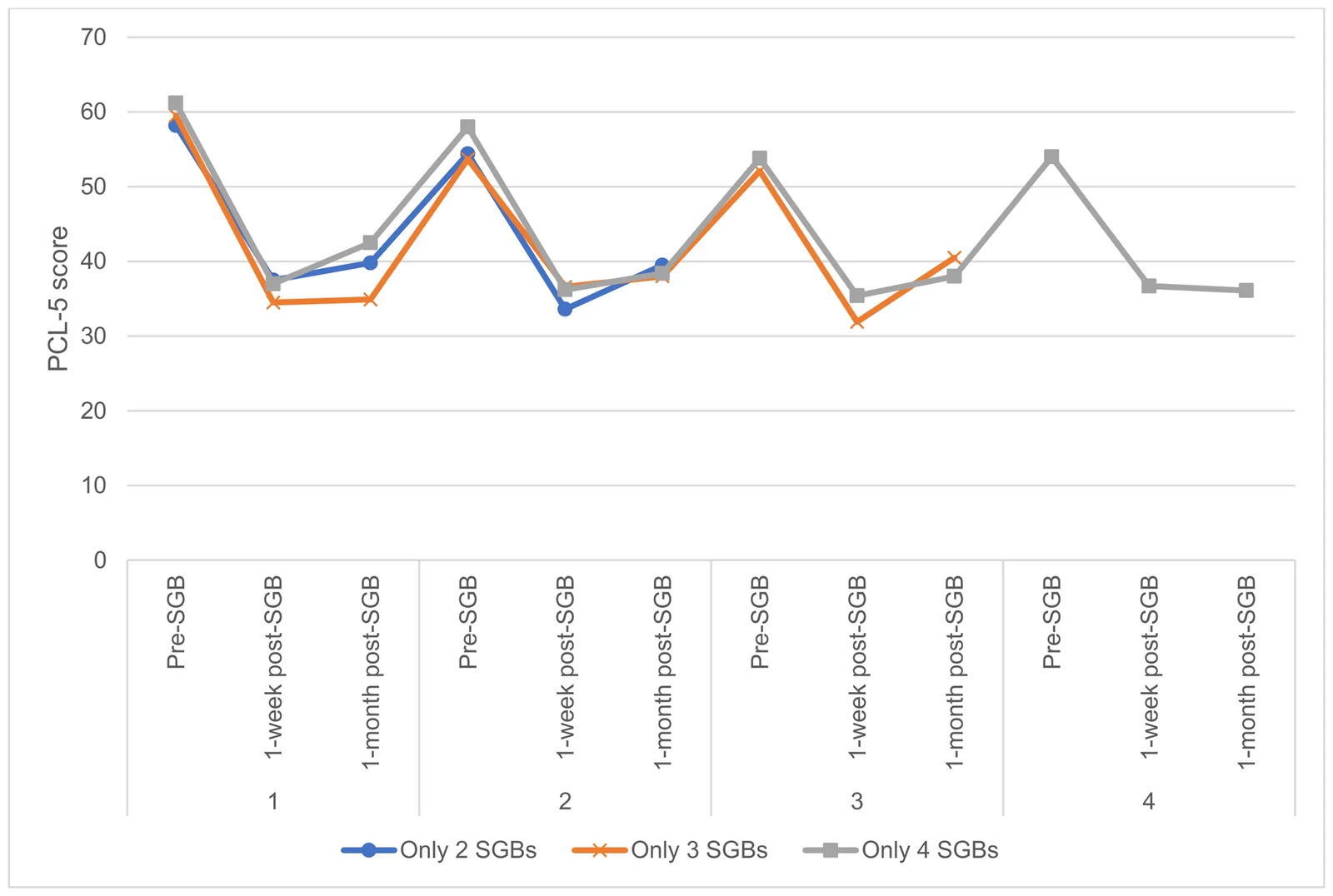
Significant baseline PTSD score reduction and trend week and month reduction with increasing number of SGBs.

Figure 2 depicts the significant reduction of PTSD symptoms pre-SGB with successive SGBs and of the non-significant trend downward at 1-week and 1-month post-SGB. The grey line—representing those who received 4+ SGBs—shows the pre-SGB PCL-5 scores move from 61.2 to 58.0 to 53.8 and 54.0 prior to each successive SGB.

### 3.3. Reliable and Clinically Significant Benefits

For all patients with complete data at both time points between pre-SGB to 1-month post-SGB, 159 of 223 (71.3%) had a ≥5-point reduction (reliable change) and 134 of 223 (60.1%) had a ≥10-point reduction (clinically meaningful change) on the PCL-5. Reliable and meaningful reductions were approximately 7% greater at the 1-week than at the 1-month post-SGB time, as expected. There were numerical but not statistically significant differences in benefit level by number of SGBs, as seen in Table 3.

### 3.4. Effect of SGB on Anxiety

These analyses were conducted for patients from each group who had complete GAD-7 data at both pre-SGB and 1-week or pre-SGB and 1-month post-SGB. On a population basis across all number of SGBs, anxiety scores were significantly reduced by 6.3 [6.1] (a 41.1% reduction) and 5.0 [5.6] (a 32.7% reduction) points at 1-week and 1-month post-SGB, respectively. Table 4 shows the number (%) of significant and clinically meaningful reductions in anxiety by number of blocks at both 1-week and 1-month post SGB. Across all number of SGBs, 51.5% and 48.3% of patients had a significant and clinically meaningful benefit. A slightly higher percentage of patients had a reduction in GAD-7 severity category (i.e., 62.5% and 58.8% at 1-week and 1-month, respectively). There were trends, but no statistically significant differences in the percentage of patients experiencing this benefit by the number of SGBs they had. There were also no significant differences in GAD-7 severity category reduction by number of SGBs delivered. However, correlations between number of SGBs and GAD-7 scores were *n* = 136, *r* = 0.18, *p* = 0.03 at 1 week and *n* = 114, *r* = 0.26. *p* < 0.01 at 1 month, showing significance in the 1-month correlation.

### 3.5. Effect of Additional Treatments

Fifty-one of sixty-one patients who received either psychotherapy, acupuncture, or biofeedback had enough baseline data to analyze vs. the whole sample who had complete data (*n* = 363). Equality of variance allowed a pooled comparison, which showed significantly lower PCL-5 scores in the additional treatment group at baseline averaged across all treatment periods, *M* = 52.6 [16.7] vs. 57.0 [14.6], *t* = 1.99, *p*_412_ = 0.047. PCL-5 scores were also lower in the additional treatment group but the comparison did not reach statistical difference at 1-week (*t* = 0.79, *p*_312_= 0.43) or at 1-month post-SGB (*t* = 1.06, *p*_271_= 0.29). There was a larger PCL-5 decrease from baseline to 1-month post-SGB in the additional treatment group (−18.8 [19.9]) than the full sample (−15.7 [16.5]) but this did not reach statistical significance (M = −18.8 [19.9] vs. −15.7 [16.5], *t* = 0.96, *p*_222_ = 0.34).

## 4. Discussion

This large case series from a cooperative clinical project evaluating the effectiveness of the Stellate Ganglion Block (SGB) for PTSD at a Veterans Administration facility showed that, on a population basis, there was a 36% and 30% reduction in PTSD symptoms from baseline to 1 week and 1 month after the first SGB, respectively. In all SPIP patients with complete data, there was a reliable symptom change in 78% of patients after 1 week and 71% after 1 month, and a clinically meaningful change in 68% after 1 week and 60% after 1 month. The absolute symptom score reduction on the PCL-5 ranged from 22.0% to 42.0%, dependent on the number of SGBs received and time from SGB (i.e., 1 vs. 4 weeks). There was also a significant effect on anxiety symptoms, with 62.5% and 58.8% having a reduction in severity category at 1-week and 1-month post-SGB, respectively. These data comport well with other case series, and in particular, the largest to date of 166 patients, showing that over 78.6% had a clinically significant benefit at 1 week and 82% at 1 month with an average reduction of 22 points on the PCL-5 [25]. That case series found a slightly larger benefit, which may be attributed to the different population (active military vs. veteran) or site-dependent effects.

There was a significant time effect on pre-SGB PCL-5 scores with a decrease from SGB 1 through 3, and there was a modest decrease trend for 1-week and 1-month post-SGB scores. This indicates an association between the number of SGBs and improvement in baseline PTSD symptoms, meaning that while symptoms eventually return some time after SGB, they may be less severe with subsequent treatments. The causal effects of this association are not clear: it may be due to additive effects of the SGB, or it may be due to other inter-SGB activities. Specifically, all patients in the program were also offered psychotherapy, acupuncture, or biofeedback after SGB and/or to continue usual care, so the positive effect on symptoms may have been due in part to these or other interventions. While the sample of those we tracked who had additional treatment and who had complete data was small, there was a signal for the added treatments being significantly associated with lower baseline scores, meaning the added treatment may have helped mitigate the severity of symptoms between SGBs. However, the absolute PCL-5 difference between those with additional treatment and those that we do not know had additional treatment was smaller than what is considered a meaningful clinical difference. A hopeful possibility is that the SGB normalized experience and reduced avoidance, allowing patients to engage more in activities than prior to treatment and to thus desensitize to PTSD-related conditioned stimuli. These data also indicate a possible association of number of SGBs with improvement in 1-week and 1-month post-SGB PTSD symptoms: the analyses show a non-significant modest downward trend of PTSD symptoms with repeat SGBs, which might be significant in a larger study with more power. Perhaps the most incisive finding is the remarkable repeatability of the SGB for PTSD and anxiety symptoms. While these findings suggest some longer-term benefit of the SGB, they most point to the consistency of effect over subsequent treatments. We suspect this repeatability applies to individuals as well as to the population as a whole.

These data and recent published studies strengthen the evidence for the effectiveness of the SGB for PTSD and anxiety symptoms in PTSD sufferers. A 2021 review concluded that the evidence for the clinical effectiveness of the SGB for PTSD is mixed [32]. This review relied heavily on a 2017 systematic review that included the first published RCT and the 2017 VA/DoD practice guidelines. It also included the 2019 positive RCT. The current study reports patient-reported benefits for both PTSD and anxiety. In 2023 Lynch and colleagues reported significant reductions in self-reported anxiety on the GAD-7 in the majority of 285 patients in a non-controlled study [33]. In an open-label randomized trial, SGBs have recently been found to potentiate the effects of psychotherapy on PTSD, anxiety, depression, and somatic symptoms when delivered prior to the therapy [34]. Behavioral health clinicians are beginning to endorse the SGB for PTSD as much or more than other therapies [35]. Evidence for the use of SGBs for a range of PTSD-related symptoms is building and more evidence from controlled trials investigating benefits on symptoms, functioning, posttraumatic growth, and biology will be helpful for determining the role of the SGB in the treatment armamentarium. Regarding clinical benefit, while the field standard for a clinically meaningful effect is a ≥10-point reduction on the PCL-5, many patients (number not clear) reported benefit with less than a 5-point reduction. These reports made clinical decision-making challenging during this project since our criteria for allowing another SGB was demonstration of benefit. Due to the frequent patient-reported benefit with less than a 5-point PCL-5 reduction, our clinical team improved decision-making by adding assessment of qualitative improvements, such as “I am more social and the family likes this” (which may be different than how they score “avoidance” on the PCL-5), or, “There is a load off of me” (often referring to a heaviness of anxiety, though the hypervigilance score on the PCL-5 only changed by 1 point). The immeasurability of benefit with the SGB is not unique in the clinical world; clinicians often see patients who say they are receiving benefit from treatment when metrics do not significantly change and vice versa, saying they are not receiving benefit when metrics do change.

From a clinical and anecdotal perspective, our clinicians frequently heard from veterans that the SGB had more “remarkable effects” than other treatments they have received. While we did not track the number or percentage of cases for which this was true, it was common to hear comments such as “I felt lighter and happier than I have since I got home” and “I haven’t been able to relax like this for years” and to see emotional expression of relief, such as tearful expressions of joy in the moments or even days and weeks after the SGB. This may be due to the rapid effect of the SGB on altering sympathetic and parasympathetic balance, something no other treatments for PTSD likely do. The SGB has been shown to “enhance” parasympathetic function (increased low to high frequency power of heart rate variability (HRV)) [36] and to have variable effects on blood pressure, heart rate, and ventricular function (depending on laterality of block), as well as on HRV and electrocardiogram changes [37]. While both the biological and psychological mechanisms of the sustained benefits of the SGB await further clarification, it is our impression informed by experience and data that the SGB reduces the rapid adrenergic burst from a conditioned trauma stimuli and provides hope that the world can once again be experienced as less threatening. A recent study in mice suggests that the SGB helps lessen fear memory consolidation by inhibition of locus coeruleus mediated norepinephrine stimulation of the basolateral amygdala [38]. The large number of communications we received from veterans expressing gratitude appears to outnumber similar sentiments expressed following other PTSD treatment modalities.

There were many benefits to infrastructure development during this project. One such benefit was gained from the limitation we note below of having incomplete data. The SPIP nurse now sends the PCL-5 and GAD-7 to patients at all time points by secure messaging with a note that these data guide our shared decision making about subsequent procedures. This change, along with making questionnaire adherence mandatory to remain in the program, has significantly improved instrument completion. A service line agreement has helped provide institutional memory about operations for this multidisciplinary treatment. Policies and procedures have developed over the project, improving patient selection, safety, standardized care, and enhanced patient-centered care.

### 4.1. Strengths and Limitations

These data come from what is most likely the largest clinical project focused on the Stellate Ganglion Block for PTSD in one of the largest healthcare systems. This project was also the result of a significant partnership between the Tibor Rubin VA Medical Center and the National Center for Healthcare Advancement and Partnerships, which is significant in helping to forward cutting-edge practices into awareness in the larger VA healthcare system. This project was not prospective research but a real-world enterprise, with the strength being that the data are likely conservative because the population is diverse and unfiltered by the many exclusions often seen in research. Conversely, this is also a limitation because we cannot say with certainty that all cases were screened with high fidelity valid techniques that occur in research protocols. However, we developed and utilized a system of evaluation using the PCL-5, GAD-7, and clinical interview, and we improved on this process over the project to have a high fidelity and systematic process for long-term care. This process will be the focus of other reports for the partnership which will be valuable if and when the SGB is more widely adopted as an evidence-based treatment. The primary limitation was the amount of missing data. We conducted 1251 SGBs in 579 unique patients in the time period of this report, yet the analyzed numbers were much smaller since we included cases from those with complete data in either two or three of the relevant time points. This limitation caused us to not report on the 4-month post-SGB data. The other obvious limitation is the non-controlled nature of this study and the use of self-report instruments. It is possible that there is a significant non-specific effect because the procedure entails considerable interaction. Each patient is first referred by a clinician, evaluated by a psychiatrist, educated by a nurse coordinator, managed on the procedure day by nurses and doctors in the procedure suite, touched and injected with care, watched for at least 30 min post-SGB, and contacted and evaluated at 1-day, 1-week, 1-month, and 3- to 4-months post-procedure, not to mention other usual care engagements. The self-report instruments are known to be valid and likely assess somewhat different domains than clinician-administered instruments.

### 4.2. Conclusions and Future Directions

The Stellate Ganglion Block seems to be an effective short-term treatment for PTSD and anxiety in a clinical setting in the VA. It may also be part of long-term symptom management using repeat SGBs at consistent intervals coupled with other care modalities. There is a signal from the data that baseline PCL-5 scores are reduced with subsequent SGBs, although causality is unclear. Patients often report high satisfaction with the SGB and its associated rapid and impactful symptom reductions. The lessons from this collaborative clinical project will be used for dissemination to other VA Medical Centers to enhance ease of start-up, fidelity, and standardization of the procedure. More research is needed to evaluate the clinical and cost-effectiveness of the SGB in contrast to or in combination with other treatment modalities. It is also important to better understand whether SGB is specific to PTSD symptoms, clusters, and constructs or if it works through a reduction of general transdiagnostic distress. A multi-site study about the efficacy and safety of the SGB for PTSD is currently ongoing with recruitment completed [39]. These data will guide questions about the need for further research and clinical initiatives to determine the place for SGB in the armamentarium of treatment for PTSD.

## Figures and Tables

**Table 1:** Demographic and SGBs administered, 21 September 2018–23 February 2024.

	*n*	Male	Female	Mean Age	Age Range
Received only 1 block	315	271 (86.0%)	44 (14.0%)	45.0	24–79
Received only 2 blocks	100	77 (77.0%)	23 (23.0%)	43.9	24–75
Received only 3 blocks	73	60 (82.2%)	13 17.8%)	44.8	27–78
Received 4+ blocks	91	75 (82.4%)	16 (17.6%)	46.6	23–76
Total unique patients	579	483 (83.4%)	96 (16.6%)	45.0	23–79

**Table 2:** PCL-5 scores [SD] and percent reduction by number of SGBs (i.e., #2–4).

SGB #		Only 2 SGBs		Only 3 SGBs		4+ SGBs		% Reduction by SGB# × Time		
		M [SD]	*n*	M [SD]	*n*	M [SD]	*n*			
1	Pre-SGB	58.2 [13.5]	95	59.5 [12.3]	70	61.2 [12.5]	88	#2	#3	#4
	1-week post-SGB	37.5 [20.5]	67	34.5 [19.0]	51	37.0 [20.4]	76	35.6	42.0	39.5
	1-month post-SGB	39.8 [20.6]	66	34.9 [17.4]	58	42.5 [20.2]	79	31.6	41.3	30.6
2	Pre-SGB	54.4 [15.8]	59	53.7 [14.7]	47	58.0 [14.1]	45			
	1-week post-SGB	33.6 [20.3]	72	36.6 [19.3]	52	36.2 [21.2]	65	38.2	31.8	37.6
	1-month post-SGB	39.5 [20.8]	67	38.0 [20.4]	54	38.4 [21.6]	68	27.4	29.2	33.8
3	Pre-SGB			52.1 [15.4]	46	53.8 [18.3]	53			
	1-week post-SGB			31.9 [19.6]	37	35.4 [21.4]	64	NA	38.8	34.2
	1-month post-SGB			40.5 [20.9]	29	38.0 [19.6]	71	NA	22.3	29.4
4+	Pre-SGB					54.0 [17.5]	59			
	1-week post-SGB					36.7 [23.6]	58	NA	NA	32.0
	1-month post-SGB					36.1 [21.4]	52	NA	NA	33.1

**Table 3:** *n* (%) reliable (≥−5 points) and clinically meaningful (≥−10 points) PCL-5 change at 1 week and 1 month after SGB by number of SGB.

	1-Week		1-Month	
	Δ-5 Points	Δ-10 Points	Δ-5 Points	Δ-10 Points
**Only 1 Block**	105/137 (76.6%)	87/137 (63.5%)	81/118 (68.6%)	67/118 (56.8%)
**Only 2 Blocks**	36/47 (76.6%)	34/47 (72.3%)	31/44 (70.5%)	25/44 (56.8%)
**Only 3 Blocks**	23/29 (79.3%)	22/29 (75.9%)	15/22 (68.2%)	13/22 (59.1%)
**4+ Blocks**	35/41 (85.4%)	29/41 (70.7%)	32/39 (82.1%)	29/39 (74.4%)
**All**	199/254 (78.4%)	172/254 (67.7%)	159/223 (71.3%)	134/223 (60.1%)

**Table 4:** *n* (%) significant symptom (≥ 6-point) and severity category reduction in anxiety on GAD-7.

	1-Week Post-SGB		1-Month Post-SGB	
	≥−6-Points	Severity Category	≥−6-Points	Severity Category
**Only 1 Block**	27/52 (51.9%)	31/52 (59.6%)	21/44 (47.7%)	23/44 (52.3%)
**Only 2 Blocks**	15/35 (42.9%)	21/35 (60.0%)	11/31 (35.5%)	17/31 (54.8%)
**Only 3 Blocks**	12/20 (60.0%)	13/20 (65.0%)	6/13 (46.2%)	7/13 (53.9%)
**4+ Blocks**	16/29 (55.2%)	20/29 (69.0%)	17/26 (65.4%)	20/26 (76.9%)
**All**	70/136 (51.5%)	85/136 (62.5%)	55/114 (48.3%)	67/114 (58.8%)

## Data Availability

The data presented in this study are available on request from the corresponding author.
